# Current Advance of Immune Evasion Mechanisms and Emerging Immunotherapies in Renal Cell Carcinoma

**DOI:** 10.3389/fimmu.2021.639636

**Published:** 2021-03-09

**Authors:** Yuli Jian, Kangkang Yang, Xiaoxin Sun, Jun Zhao, Kai Huang, Abdullah Aldanakh, Zhongyang Xu, Haotian Wu, Qiwei Xu, Lin Zhang, Chunyan Xu, Deyong Yang, Shujing Wang

**Affiliations:** ^1^Department of Biochemistry and Molecular Biology, Institute of Glycobiology, Dalian Medical University, Dalian, China; ^2^Department of Urology, First Affiliated Hospital of Dalian Medical University, Dalian, China

**Keywords:** renal cell carcinoma, tumor immune evasion, immunotherapy, immune checkpoint, tumor microenvironment

## Abstract

Renal cell carcinoma is a highly heterogeneous cancer group, and the complex microenvironment of the tumor provides appropriate immune evasion opportunities. The molecular mechanism of immune escape in renal cell carcinoma is currently a hot issue, focusing primarily on the major complex of histocompatibility, immunosuppressive cells, their secreted immunosuppressive cytokines, and apoptosis molecule signal transduction. Immunotherapy is the best treatment option for patients with metastatic or advanced renal cell carcinoma and combination immunotherapy based on a variety of principles has shown promising prospects. Comprehensive and in-depth knowledge of the molecular mechanism of immune escape in renal cell carcinoma is of vital importance for the clinical implementation of effective therapies. The goal of this review is to address research into the mechanisms of immune escape in renal cell carcinoma and the use of the latest immunotherapy. In addition, we are all looking forward to the latest frontiers of experimental combination immunotherapy.

## Introduction

Renal cell carcinoma (RCC) is one of the urinary system malignant tumors that accounts for 3% of all new cases of cancer in females and 5% in males with an incidence of about 400,000 cases worldwide, and it's one of the world's 10 most common cancers (1). RCC is a heterogeneous group of cancers arising from renal tubular epithelial cells, consisting of different subtypes, clear cell RCC (ccRCC) is the most frequent, accounting for about 75%, followed by papillary (pRCC) 15%, chromophobe (chRCC) 5%, and unclassified RCC (uRCC) 4% (2). Following the classification of renal cell carcinoma by the World Health Organization in 2016, new and emerging provisional renal entities have been defined by scientists; this significantly deepens the understanding of the immunohistochemistry, morphology and molecular features of renal tumors (3).

The body exerts an anti-tumor effect in normal circumstances through the immune system, if the role of immune surveillance decreases, this may lead to malignant tumor development, which requires a number of mechanisms of immune evasion. Tumor immune escape emerged from the immune surveillance hypothesis suggested by Burnet (4). They claim that the immune system of the body will control mutant “non-self” cells, which can be explicitly removed to preserve the integrity of the microenvironment of the body through cellular immune mechanisms. However, when mutant cells escape surveillance of the immune system under the influence of various internal or external factors, the tumor will continue to proliferate and deteriorate. Tumor immune escape includes three processes: immune elimination, immune equilibrium and immune escape. Multiple aspects of the immune escape mechanisms of renal cell carcinoma include the alteration of tumor autoantigenicity and the development of immunosuppressive microenvironments. The factors listed above directly or indirectly affect tumor cells or immune cells and eventually contribute to the incidence of immune evasion, so understanding the immune escape mechanisms of renal cell carcinoma is of great importance for the treatment of renal cell carcinoma, especially immunotherapy that already has made considerable progress in recent years. Existing immunotherapy strategies for RCC include cytokines, vaccines, monoclonal antibodies, immunocheckpoint inhibitors (ICI), chimeric antigen receptor (CAR) modified immune cells therapy and combination immunotherapy. In this review, the possible molecular escape mechanisms for renal cell carcinoma will be addressed, as well as the theory and clinical application of immunotherapy.

## Mechanisms of Immune Evasion in Renal Cell Carcinoma

While some anti-tumor immune response may be produced by the body's immune system, many tumors still develop in the body and even cause host death, indicating that the tumor may elude the host immune system or prevent the body from generating effective anti-tumor immune response by some mechanisms. Most tumors can develop or metastasize under the supervision of the immune system, which cannot be distinguished from the incidence of immune escape. In the case of extremely heterogeneous cancers, such as RCC, changes in tumor autoantigenicity and complex immune microenvironment provide favorable conditions for tumor immune escape. The following aspects are unique molecular mechanisms: abnormality in the presentation of antigen and processing mechanisms, in particular the abnormal expression of class I molecules of the major histocompatibility complex (MHC), which enable tumor cells to avoid immune surveillance (5). RCC cells may induce cytokine expression in the tumor microenvironment (TME) in the phase of tumorigenesis and growth, leading to an immunosuppressive tumor state and promoting immune escape. Tumor-related immunosuppressive cells play an “accomplice” role in tumor growth, metastases and invasion. At the same time, immune cell apoptosis caused by various parameters is also of great significance for immune surveillance and escape.

### Abnormal Expression of MHC Class I Molecules

Under immune surveillance, renal cell carcinoma cells survive, requiring immune escape to expand further. While molecular mechanisms are still difficult to elucidate, the abnormal expression of MHC class I molecules in many cancers, including renal cell carcinoma, provides a key strategy for immune evasion (5). In tumor cells, the down-regulation of MHC class I molecules helps them to avoid detection by CD8^+^ T cells, meanwhile changes in the processing pathway of classical and non-classical human leukocyte antigen (HLA) and antigen presentation provide multiple pathways for renal cell carcinoma cells to weaken the immune responses of the body, resulting in more tumor growth and host immune surveillance evasion (6).

#### Classical HLA-I Molecules

HLA-I molecules are transmembrane glycoproteins, which contain polymorphic heavy chains (α chain; 45 kDa) and non-polymorphic light chains (β2-microglobulin; 12 kDa) distributed on the surface of different nucleated cells and platelets. As a result, HLA-I expression defects are usually related to disease progression and poor prognosis, such as bladder cancer (7), kidney cancer (8), lung cancer (9, 10) and other solid tumors. Molecular defects of HLA-I are shown as complete loss or down-regulation, haploid loss, locus loss, allelic loss, compound phenotype, interferon unresponsiveness, classical HLA-A, -B, and -C down-regulation molecules, and the presence of non-classical HLA-E molecules (5, 11). However, there is no knowledge about alterations in the expression of HLA-I molecules in renal cell carcinoma, this seven identified phenotypes found in different types of tumors (12, 13). Romero et al. (14) found that 39% of tumor lesions in renal cell carcinoma displayed partial loss of HLA-I molecules, and 6% of the tumors showed complete loss of HLA-I molecules. Primary renal cell carcinoma showed a low degree and frequency of HLA-I expression changes. This abnormal shift is due to the lack of expression of components in antigen processing machines, in particular transport proteins associated with antigen processing (TAP) 1, TAP 2 and low molecular weight proteins (LMP) 2, 7, and 10 (15). As a consequence, the abnormal antigen processing machine portion of HLA-I molecules in renal cell carcinoma appears to represent the RCC's immune escape mechanism, rather than the HLA-I expression. It reduces the immune surveillance capability of CD8^+^ cytotoxic T lymphocytes and/or natural killer (NK) cells and promotes further tumor growth. Furthermore, a study showed that interferon-γ (IFN-γ) stimulates cell lines of human renal cell carcinoma to increase transcription and protein levels of TAP 1 and LMP 2 (16), indicating that co-transcriptional upregulation of IFN-γ-mediated TAP 1 and LMP 2 expression can lead to increased immune system recognition of renal cell cancer cells, thereby avoiding immune escape.

#### Non-classical HLA-I Molecules

HLA-G, as a non-classical molecule of MHC-I, has minimal distribution characteristics of tissues. The biological functions and clinical importance of HLA-G have been intensively studied in recent decades. It is now widely accepted that HLA-G is an important marker of immune tolerance in cancer cell immune escape and is closely related to the progression and prognosis of cancer patients. HLA-G expression in multiple tumors shows strongly contradictory results (17). HLA-G is not expressed in all forms of renal epithelial cell tumors, but is restricted to clear cell carcinoma (18). Besides, the expression of HLA-G is found to be up-regulated in different solid tumors and hematological diseases (19, 20). HLA-G is present in tumor tissues and/or infiltrating cells (including predominantly monocytes, macrophages and lymphocytes) but not in normal tissues (21). Studies have shown that most tumor cell lines, especially malignant melanomas, are positive for HLA-G mRNA (22, 23), but some reports disagree (24).

Like melanoma, while renal cell carcinoma has some immunogenicity, tumor cells can still develop and may be associated with the absence of anti-tumor reactions. The abnormal tumor cell expression of HLA-G molecules is part of the strategy for evading host immune surveillance. HLA-G molecules play a key role in establishing and maintaining immune tolerance by inhibiting the function of immune cells (21), and their direct binding to inhibitory receptors facilitates this inhibition. Studies have shown that HLA-G mRNA and protein expression are present in renal cell carcinoma lesions, but there is no expression in normal renal epithelial cells, but there is no correlation between HLA-G expression and clinical parameters, such as tumor stage, grade, and patient age. Their data indicated that, for the first time, HLA-G-expressing RCC cell lines were less responsive to CTL, lymphokine activated killer cells, and NK-mediated cytotoxicity than HLA-G^−^ normal kidney cells (25). These phenomena suggest that the expression of HLA-G in RCC cells contributes to impaired immune recognition of CTL, NK, and lymphokine activated killer cells, which may play a role in avoiding immune surveillance.

Another non-classical HLA molecule is HLA-E, which, like HLA-G, has immunosuppressive properties, and studies have found higher levels of HLA-E transcripts detected in all RCC cell lines and tumor lesions compared to standard renal epithelium. The expression of HLA-E was not associated with the level of infiltration of immune cells by CD3^+^, CD4^+^, CD8^+^, and FOXP3^+^, but was negatively correlated with infiltrating CD56^+^ cells (26). This indicates that overexpression of HLA-E decreases the immunogenicity of cells with renal cell carcinoma and may therefore play a role in promoting immune surveillance evasion.

### Expression of Immunosuppressive Cytokines

There is a complex relationship between tumor cells and the immune system. Cytokines formed by tumor cells or immune microenvironment play a key role in the interaction in which immunosuppressive cytokines, such as interleukin-10 (IL-10), prostaglandin E2 (PGE2), transforming growth factor-β (TGF-β), and vascular endothelial growth factor (VEGF) are involved in cancer growth, proliferation and invasion. It is precisely because of the production of these immunosuppressive cytokines that the tumor cells represent the immunosuppressive state and then escape immunosuppressive surveillance. Understanding the impact and molecular mechanisms of immunosuppressive cytokines on tumors may therefore target cytokines to improve promising immunotherapy for renal cell carcinoma therapy.

#### Interleukin-10

IL-10 (37 kDa) contains 178 amino acids, including a 160 amino acid secretory protein and an 18 amino acid signal peptide. Some studies have shown that IL-10 is generated primarily by regulatory T cells (Tregs) and other immunosuppressive cells (27). IL-10 is a T cell proliferation and cytokine response inhibitory cytokine that plays an important role in inflammatory regulation and in directing adaptive immune responses. It often seems to be contradictory and is still the subject of research because of the high diversity of IL-10.

Since IL-10 is generally recognized as an immunosuppressive cytokine, IL-10 is known to be able to facilitate tumor immune escape by reducing the antitumor immune response in the tumor immune microenvironment. It has been stated that IL-10 expression in cervical cancer can induce an immunosuppressive environment by up-regulation of HLA-G expression and down-regulation of HLA I expression (28). Furthermore, the researchers also find that tumor cells themselves can produce IL-10 (29, 30), while renal cell carcinoma can induce monocytes to produce IL-10 and control the expression of molecules on the surface of cells presenting antigen (31). The responses of infiltrating tumor lymphocytes have been reported to be correlated with the prognostic value and therapeutic value of different cancers (32). Cai et al. (33) focused on cells that can produce IL-10 in tumor-infiltrating lymphocytes in renal cell carcinoma. Inverse associations were found between the frequency of IL-10-producing B cells and the pro-inflammatory cytokine-producing T cells, as well as the proliferation of T cells, thus it is hypothesized that in renal cell carcinoma, IL-10-producing B cells can contribute to T cell immunosuppression.

#### Prostaglandin E2

As we all know, the immune microenvironment provides a variety of tumor-promoting factors that can not only promote tumor development, but also establish immunosuppressive conditions that enable tumor cells to resist immune surveillance and induce tumors to evolve freely. The most important and active final product of the COX-2 pathway is PGE2, which is known to be biologically lipid with evidence of immunosuppressive properties (34, 35). Fibroblasts and some immune cells in TME have been shown to express high levels of COX-2 and PGE2 that been up-regulated in a variety of cancers and a certain correlation exists between PGE2 expression and tumor progression (36–38).

Verratti et al. (39) assessed the PGE2 content of human renal parenchymal and renal cell carcinoma and found that the RCC expression of PGE2 was higher than that of normal renal tissue, unrelated to renal cell carcinoma size, fuhrman grade, pathologic tumor lymph node, and histologic subtype metastasis. Furthermore, there was no substantial difference between clear and non-clear cell types in PGE2 expression, suggesting that PGE2 had a tumor-promoting effect irrespective of the histological origin of renal cell carcinoma. Similarly, immunohistochemical findings showed that COX-2 was highly expressed in carcinoma of renal cells and that the degree of COX-2 expression was related to the grade of the tumor and the pathological stage (40, 41). It is hypothesized that overexpression of PGE2 and COX-2 in renal cell carcinoma could be linked to pathological changes in renal cell carcinoma, which may be useful biomarkers in RCC. Report on lung cell carcinomas prove that COX-2 derived PGE2 plays a significant role in the transformation of regulatory T cells (42), and researchers have found that COX-2 derived PGE2 in renal cancer can transform CD4^+^FOXP3^−^ T cells into CD4^+^CD25^+^FOXP3^+^ regulatory T cells to escape the immune system (43). Renal cell carcinoma has been reported to induce the production of PGE2 and T-helper type 2 (Th2) cytokines in peripheral blood monocytes, which inhibits host anti-tumor immune responses and therefore reduces the expression and endocytosis of cell surface molecules involved in antigen presentation (44). Changes in COX-2 and PGE2 in renal cell carcinoma suggest changes in the immune microenvironment of the tumor.

#### Transforming Growth Factor-β

Inflammation, tissue repair and embryonic development have been the vital biological functions of TGF-β (45–47), but TGF-β has been found to play an important role in regulating cell growth, development, differentiation and immune function in recent years (48, 49). Although TGF-β has a tumor suppressive effect by inhibiting cell cycle progression and promoting early cancer cell apoptosis, it promotes tumor development, metastases, and inhibits host anti-tumor immunity in advanced stages via encouraging transformation from epithelial cells to stromal cells, and by encouraging immune cell phenotyping to tumor support (50).

The immunosuppressive TGF-β pathway in many solid tumors is associated with poor prognosis especially in advanced tumors (51). Renal cell carcinoma causes an immunosuppressive phenotype in peripheral blood, which is characterized by up-regulation of TGF-β expression that could stimulate intratumoral angiogenesis and inhibit humoral and cellular immunity (52), meanwhile TGF-β1 down-regulates the expression of MHC-II and B7-1 in primary mouse renal tubular epithelial cells and affects antigen presentation thereby inhibiting T cell-mediated immune responses (53). One study mentioned that, TGF-β controls the adaptive immune response by directly promoting the production of regulatory T cells and inhibiting the production and function of effector T cells and DCs (54). By inhibiting the activity of NK cells, TGF-β also regulates the innate immune response (55). It should be noted that some studies have found that TGF-β secretion in B cells is linked to IL-10 expression (56), indicating that complex network functions among immunosuppressive cytokines secreted by carcinoma of renal cells may occur.

#### Vascular Endothelial Growth Factor

VEGF is mostly a 34–42 kDa protein produced by tumor cells that stimulates endothelial cell proliferation and angiogenesis (57). The fact that Von Hippel-Lindau (VHL) mutations occur in most ccRCC patients is a significant characteristic of clear cell RCC, resulting in alterations in the hypoxia pathway leading to increased VEGF expression, increased angiogenesis and immune suppression (58). VEGF is the major inhibitory cytokine in TME and its expression level is closely linked to the clinical prognosis of tumor patients (59). Immunohistochemical findings in cancer cells have shown that there is a negative association between DC density and VEGF expression (60), indicating that VEGF inhibits DC function in TME, and VEGF expression may be linked to tumor progression and poor prognosis, not only because VEGF induces tumor angiogenesis, but also protects tumor cells from attacks by the immune system.

Previous studies have shown that VEGF plays a major role in DC differentiation and inhibits the maturation of immature DC (61–63). Therefore, use of inhibitors targeting VEGF or its receptors and inhibition of VEGF signal transduction can effectively improve the differentiation and maturation of DC, but they are not sufficient to improve the immune response (64). In reality, immature or incompletely mature DC can mediate tumor immune tolerance and induce T cell anergy or the proliferation of regulatory T cells (65). Therefore, both the changes of VEGF itself and the immune cells controlled by VEGF may be the causes of immune escape. Likewise, VEGF also has a certain effect on the immune system of mature DC. The RhoA-cofilin1 signal transduction pathway regulated by VEGF receptor 2 impairs the migration capacity and immune function of mature DC (66). It is proposed that this pathway should be blocked by using anti-tumor immunotherapy based on DC, which is of great significance for understanding the mechanisms of immune escape and the application of subsequent immunotherapy.

### Generation of Immunosuppressive Cells

Regulatory T cells, regulatory B cells (Bregs), myeloid-derived suppressor cells (MDSCs), and tumor-associated macrophages (TAMs) can induce immunosuppressive responses during tumor immune escape, in addition to the two possible mechanisms mentioned above. Tregs, Bregs, MDSCs, and TAMs-mediated immune escape pathways have not yet been thoroughly studied. The relevant studies currently focus primarily on the factors provided by immunosuppressive cells and their pathways of signal transduction, as well as on the interaction of immunosuppressive cells with other immune cells. These variables can play an immunosuppressive role and induce immune escape to occur.

#### Regulatory T Cells

Regulatory T cells are a heterogeneous cell population, primarily divided into CD4^+^ T cell subsets and CD8^+^ T cell subsets. Conceptually, CD4^+^ regulatory T cells are classified into three groups. The thymus is the source of CD4^+^CD25^+^FOXP3^+^ T cells and is known to be naturally occurring regulatory T cells (Tregs) (67). CD4^+^IL-10^+^FOXP3^−^ T cells, known as T-type regulatory cells 1, can interact with exogenous antigens in a number of ways, which not only inhibit the adaptive immune response, but also play a role in the regulation of the innate immune response (68). CD4^+^TGF-β^+^ T cells called T_H_3 cells are induced by oral tolerance (69). Regulatory T cells can suppress immune responses by interacting with other immune cells or by producing immunosuppressive cytokines, so they play a vital role in maintaining immune homeostasis and mediating peripheral tolerance (70, 71).

Clinical data show that CD4^+^CD25^+^FOXP3^+^ regulatory T cells are over-expressed in RCC patients' peripheral blood, which directly affects patients' T cell response (72). The rise in Tregs in tumors is related to the poor survival of many patients with solid tumors, including renal cell carcinoma (73), breast cancer (74), gastric cancer (75), and ovarian cancer (76). Therefore, tracking the role of Tregs in peripheral blood in patients with renal cell carcinoma is helpful in understanding the immune response of the antitumor and predicting the impact of immunotherapy. Indoleamine 2,3-dioxygenase (IDO) is an important negative regulator of the immune system that has gained a great deal of attention in recent years. The interaction between IDO and CD4^+^CD25^+^ regulatory T cells can play a key role in tumor immune escape, but is still controversial. However, IDO in endothelial cells of renal cell carcinoma can reduce the flow of tryptophan from the blood to the tumor or produce tumor-toxic metabolites, thereby limiting tumor growth and contributing to patient survival (77). Xu and his team have found that IL-23 secreted by macrophages enhances the immunosuppressive role of Tregs in glutamine-addicted clear cell renal cell carcinoma to prevent the cytotoxicity of CD8^+^ T cells, which in turn is associated with immune escape (78).

#### Myeloid-Derived Suppressor Cells

Myeloid-derived suppressor cells exist in most cancer patients that contribute to immunosuppressive TME, so they play a key role in promoting tumor growth, metastasis, and they may even reduce the effectiveness of immunotherapy (79–82). Accumulating evidence demonstrates that a number of patients with renal cell carcinoma do not respond to immune checkpoint inhibitor therapy, and there may be severe immunosuppression, which is mediated partly by MDSCs (83). This implies that the frequency and immunosuppressive function of MDSCs in cancer patients can be used as a predictor of patient response to treatment. MDSCs have aroused great interest because they have a biological role in tumor-mediated immune escape by inhibiting anti-tumor immune responses. In the context of cancer, TME allows MDSCs and Tregs to be very close, so there is a lot of crosstalk to regulate two immunosuppressive cell populations. Indeed, Ghiringhelli et al. (84) have shown that immature myeloid cells induced by tumor progression selectively promoted the proliferation of Tregs in a TGF-β-dependent manner *in vivo*. Huang et al. (85) also proved that MDSCs induced the development of Tregs cells in cancer patients and tumor-bearing mice, and the induction of Tregs depended on IL-10 and IFN-γ secreted by MDSCs, which can significantly induce the anoreactivity of effector immune cells. The immune escape mechanisms mediated by MDSCs also include nitrating lymphocyte-specific protein tyrosine kinase (LCK) inhibiting T cell activation (86), inducing T cell dysfunction through arginase 1 (Arg1) metabolism depletion of arginine (87), and tumor-derived extracellular vesicles (EVs) inducing MDSCs production, recruitment and activation (88).

In addition to the immune escape mechanisms, MDSCs can dynamically reshape the TME through the production of angiogenic factors and metalloproteinases. They can also help to create a pre-metastatic environment and promote the epithelial-to-mesenchymal transfer of tumor cells to maintain tumor progression (89). The study found that the overall MDSC in patients with renal cell carcinoma periphery was increased relative to standard controls, and the polymorphonuclear MDSC (PMN-MDSC) was associated with the grade of cancer, suggesting that PMN-MDSC could be a prognostic marker. Furthermore, the blockade of interleukin-1β (IL-1β) resulted in decreased parenchymal PMN-MDSC, peripheral PMN-MDSC, monocytic MDSC and inhibited tumor progression (82). This indicates that anti-IL-1β could be a possible strategy for attacking MDSC immune inhibitors. Exosomes formed by tumor cells have an important role to play in immunosuppression. Zhang et al. (90) found that the interactive exosomal HSP70 and MDSCs decide the suppressive activity of MDSCs through Stat3 phosphorylation. PGE2 induced by renal cell carcinoma induces Arg1 expression in MDSCs, which depletes arginine in the microenvironment and leads to significant molecular changes in T cells, including loss of T cell.

#### Tumor-Associated Macrophages

Tumor-associated macrophages are an important part of tumor stroma. They originate from blood monocytes attracted by chemokines and cytokines produced by tumor cells, and develop into effective tumor supporting cells under the guidance of TME, which can promote tumor progression and inflammation. Hypoxia is a key feature in the microenvironment that is actively involved in tumor progression. Osinsky et al. (91) studied the effects of hypoxia and hypoxia-related factors on tumor progression and survival, and they found that when hypoxia occurred, the expression of VEGF was up-regulated, the number of TAM increasing, and the matrix metalloproteinases activity increasing, which are negatively correlated with the overall survival (OS) rate of patients. We can know that hypoxia-related signals activated in tumors promote tumor progression through macrophage recruitment, extracellular matrix remodeling and new angiogenesis.

Studies have revealed that macrophages infiltrating renal cell carcinoma secrete immunosuppressive cytokines IL-10 and cell chemokine ligand 2 (a proinflammatory chemokine). TAM also promotes immune escape by inducing the production of tolerant T cells and up-regulating the expression of cytotoxic T lymphocyte antigen 4 (CTLA-4) in autologous T cells (92). Chen et al. (93, 94) studied B7-homolog 3 (B7-H3) and B7-homolog 4 (B7-H4) expressed on macrophages in lung cancer stroma and found that lung cancer cells and TAM-related B7-H3/B7-H4 were strong inhibitors of T cell effect, both of which acted in the form of costimulatory. The expression of B7-H3/B7-H4 in renal cell carcinoma is associated with poor clinical and pathological features, including tumor size, stage and grade, and B7-H3/B7-H4 expression is also associated with poor prognosis (95, 96). It can be concluded that B7-H3 and B7-H4 may be useful prognostic markers in patients with RCC. The membrane-bound B7-H3/B7-H4 induced by TAM-tumor cell interaction represents a new mechanism of immune escape, which links the pro-inflammatory response with immune tolerance in tumor environment.

#### Regulatory B Cells

Regulatory B cells are immunosuppressive cells that support immune tolerance and secrete anti-inflammatory cytokines, including IL-10, IL-35, and TGF-β. Although the functions of Tregs, MDSCs, and TAMs in anti-tumor immune response have been extensively studied, the emerging role of B cells has been given more and more attention by researchers. Currently, there are a range of regulatory B cell phenotypes known that play an immunosuppressive function in autoimmune diseases and cancers (97). In addition to Tregs, the Bregs also have immunomodulatory and/or immunosuppressive roles, which may play a key role in the regulation of tumor immune response (98, 99). IL-10 is the most common cytokine linked to Bregs in tumors and autoimmune diseases (100–102). IL-10-producing B cells, namely regulatory B cells, cause T cells to differentiate into Tregs and facilitate the spread and activation of Tregs, mediating tumor immune escape. Study found the same phenomenon in tongue squamous cell carcinoma. CD19^+^IL-10^+^ regulatory B cells induced resting CD4^+^ T cells to transform into CD4^+^FOXP3^+^ regulatory T cells and predicted a worse prognosis (103).

Tumor-induced Bregs are recruited into the immune microenvironment and are an important mechanism for avoiding protective immunity and promoting metastatic growth in tumor cells. Olkhanud et al. (104) found that in the mouse model of breast cancer, Bregs induced CD25^+^CD69^+^ tumors stimulated lung metastases by inducing TGF-β-dependent differentiation of FOXP3^+^ Tregs. Interestingly, tumor-induced Bregs express high levels of CD80 and CD86, suggesting that CD80 and CD86-mediated contacts between Bregs and their target cells are essential for suppressing T cell response and Bregs-induced Tregs differentiation. However, there are very few reports on Bregs in renal cell carcinoma metastases. In sum, the important role of Bregs in renal cell carcinoma is to induce the development of regulatory T cells through the production of IL-10, which then plays a role in immune escape. Whether other cytokines generated by Bregs or immune cells in renal cell carcinoma that contribute to the immune escape mechanism need further clarification.

### Occurrence of Apoptosis of Immune Cells

A transmembrane protein belonging to the tumor necrosis factor/nerve growth factor receptor superfamily is Fas (also known as CD95/Apo-1). It transmits apoptosis signals to susceptible cells once activated by its natural ligand, FasL (105). While Fas signal from FasL can induce tumor cell apoptosis and inhibit tumor growth, studies have also shown that Fas signal in the presence of FasL can promote tumor growth (106, 107). These findings indicate that the Fas/FasL system plays a complex role in the induction of apoptosis. Cai and colleagues (108) found that activated T cell exosomes release FasL in mouse models of melanoma and lung cancer, which increases the expression of matrix metalloproteinase 9 via the Fas signaling pathway, thus improving the invasive potential of the tumor, thus providing new perspectives for understanding tumor evasion from immune surveillance. In addition, studies have shown that Fas/FasL signaling pathways in mouse H22 hepatoma cells are involved in immune escape by inducing Jurkat T cell apoptosis. Decreasing FasL expression in tumor cells can reduce the apoptosis rate of immune cells and further block the apoptosis signal pathway of immune cells by preventing tumor necrosis factor-induced apoptosis, which may boost the survival rate of immune cells (109). Available evidence suggests that renal cell carcinoma cells can express FasL, and it is believed that increased expression of FasL in RCC is one of the important mechanisms for avoiding immune attacks (110).

Immune cells (such as T cells) are the central elements in the immune response, but in the presence or involvement of certain molecules, immune cell apoptosis cannot play a role in destroying tumors, thus cancer escape immune system. B7-H1, a member of the B7 costimulatory molecule family, is expressed in many human cancers and B7-H1 facilitated antigen-specific T cell apoptosis *in vitro* or in mouse P815 tumor models was primarily mediated by one or more receptors other than the programmed cell death protein-1 (PD-1) (111). CD70, a cytokine overexpressed in renal cell carcinoma, promotes lymphocytes apoptosis by interfering with its CD27 receptor and intracellular SIVA protein binding, rendering it difficult for patients to develop an efficient lymphocyte-mediated anti-tumor response (112). The above findings indicate that the mechanism of tumor-induced T cell apoptosis is receptor-dependent, so researchers turn their attention to soluble tumor-derived factors and predict whether T cell apoptosis can be independently induced by the receptor. Kudo et al. (113) found that gangliosides in the RCC cell line supernatant (SK-RC-45) were involved in tumor-induced T cell apoptosis through reduction of Bcl-2 and Bcl-XL expressions in lymphocytes, the release of cytochrome c and the activation of caspase in mitochondria simultaneously. In summary, renal cell carcinoma tumors can induce T cell apoptosis by synthesizing products of both receptor-dependent and receptor-independent pathways, and by activating both independent apoptotic pathways.

**Figure 1:**
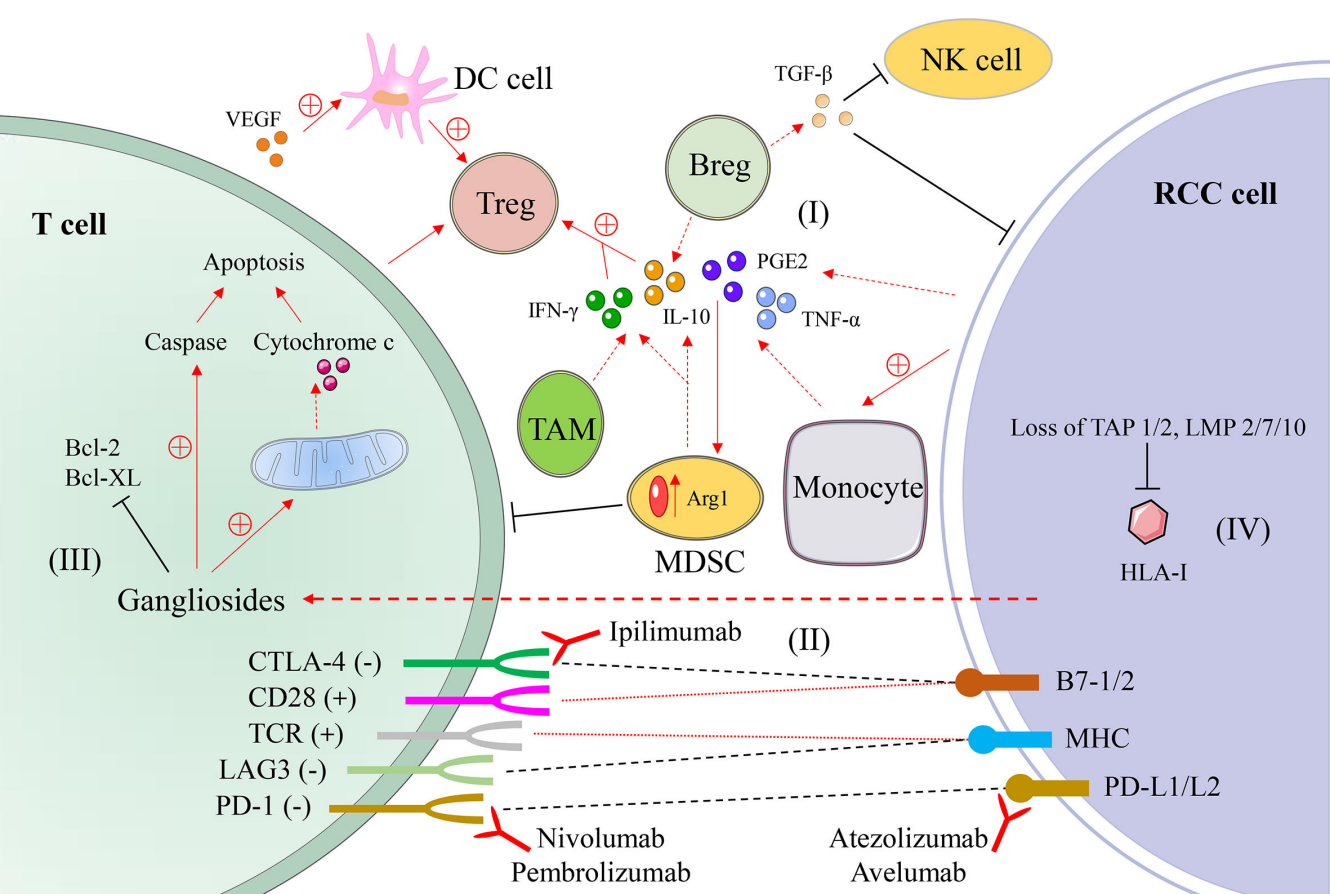
A schematic diagram of mechanisms of immune evasion in renal cell carcinoma. (I) Immunosuppressive cells and their secreted cytokines, such as interleukin-10 (IL-10), prostaglandin E2 (PGE2), and transforming growth factor-β (TGF-β) secreted by myeloid-derived suppressor cell (MDSC) and other cells. (II) The interaction of T cells and tumor cells and the mode of action of monoclonal antibodies. (III) Gangliosides secreted by renal cell carcinoma cells promote T cell apoptosis. (IV) The absence of components related to antigen processing leads to a decline in antigen presentation function. RCC, renal cell carcinoma; DC, dendritic cell; NK, natural killer cell; Treg, regulatory T cell; Breg, regulatory B cell; TAM, tumor-associated macrophage; IFN-γ, interferon-γ; TNF-α, tumor necrosis factor-α; HLA, human leukocyte antigen; TAP, transport protein proteins associated with antigen processing; LMP, low molecular weight protein; CD28, cluster of differentistion 28; CTLA-4 (CD152), cytotoxic T-lymphocyte associated protein 4; TCR, T cell receptor; LAG3 (CD223), gene 3 activation of lymphocyte; PD-1 (CD279), programmed cell death protein 1; B7-1/2 (CD80/CD86); MHC, major histocompatibility complex; PD-L1/L2 (CD274/CD273), programmed cell death protein ligand 1/2.

There are a variety of explanations for immune escape and tumor development in renal cell carcinoma. Table 1 summarizes the basic mechanisms of immune escape, including the expression of HLA-I molecules and changes in cytokines. A variety of immunosuppressive cytokines and immunosuppressive cells in TME of renal cell carcinoma generate inhibitory conditions to inhibit congenital or adaptive immune responses, creating conditions conducive to tumor escape (Figure 1).

**Table 1:** Immune escape mechanisms in renal cell carcinoma.

**Renal cell carcinoma cells**	**Tumor microenvironment**				
	**Immunosuppressive cytokines**	**Metabolic enzymes**	**Immunosuppressive cells**	**Immune checkpoints**	**Others**
Classical HLA-I molecules↓	IL-10↑	COX-2↑	Tregs↑	PD-1	Hypoxia↑
Non-classical HLA-I molecules↑	PGE2↑	IDO↑	MDSCs↑	PD-L1/2	Cytochrome c↑
FasL↑	TGF-β↑	LCK↑	TAMs↑	CTLA-4	
PD-L1/2	VEGF↑	Arg1↑	Bregs↑	CD28	
				LAG3	
				TIM-3	

*TME, tumor microenvironment; HLA, human leukocyte antigen; FasL, Fas ligand; PD-L1/2, programmed cell death protein ligand 1/2; IL-10, interleukin-10; PGE2, prostaglandin E2; TGF-β, transforming growth factor-β; VEGF, vascular endothelial growth factor; COX-2, cyclooxygenase-2; IDO, indoleamine 2, 3-dioxygenase; LCK, lymphocyte-specific protein tyrosine kinase; Arg1, arginase 1; Tregs, regulatory T cells; MDSCs, myeloid-derived suppressor cells; TAMs, tumor-associated macrophages; Bregs, regulatory B cells; PD-1, programmed cell death protein 1; CTLA-4, cytotoxic T lymphocyte antigen 4; CD28, cluster of differentistion 28; LAG3, gene 3 activation of lymphocyte; TIM-3, T cell immunoglobulin mucin molecule 3; ↑, increase; ↓, decrease*.

## Immunotherapies for Renal Cell Carcinoma

**Figure 2:**
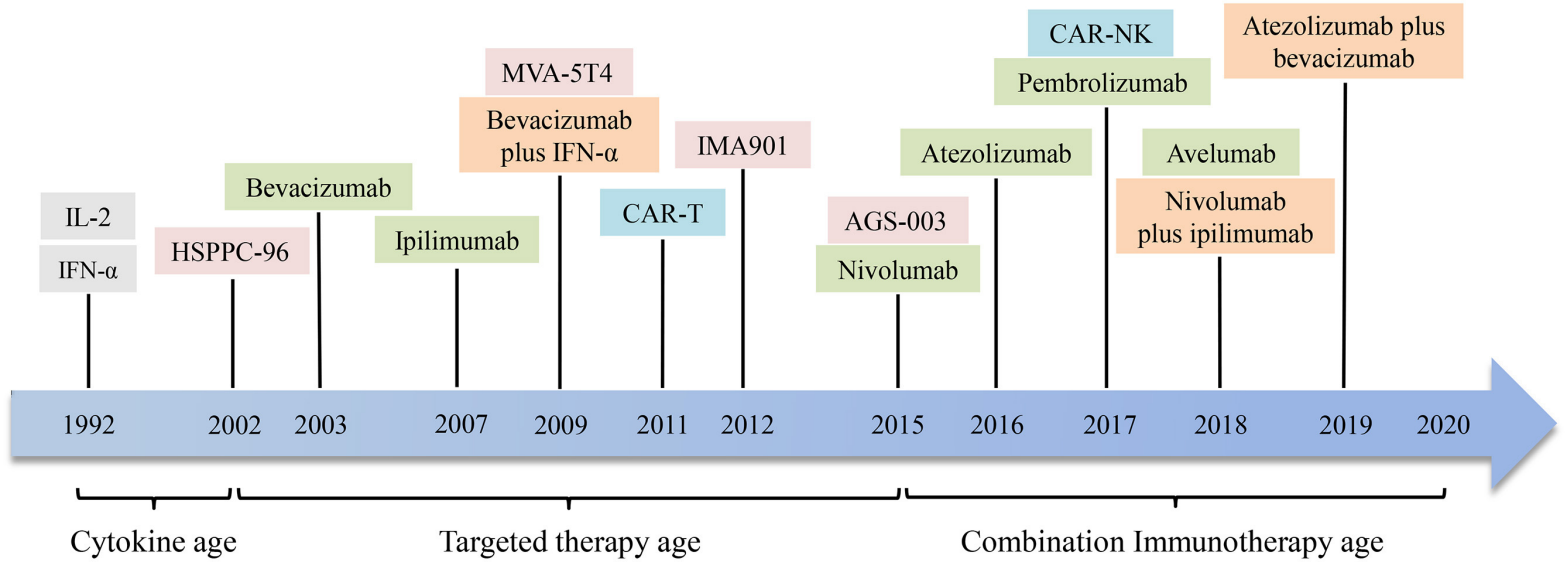
The development history of immunotherapies in renal cell carcinoma. In 1992, interleukin-2 (IL-2) and interferon-α (IFN-α) were used to treat renal cell carcinoma. Then through the era of targeted drug therapy, drugs targeting specific antigen targets and immune checkpoints have shown considerable clinical efficacy. In recent years, the application of combined immunotherapy has improved the remission rate and prolonged survival of patients with renal cell carcinoma. It is hoping that more drugs and combination immunotherapies will appear in the future. HSPPC-96, heat shock protein peptide complex 96; CAR-T, chimeric antigen receptor T cell; CAR-NK, chimeric antigen receptor NK cell.

**Figure 3:**
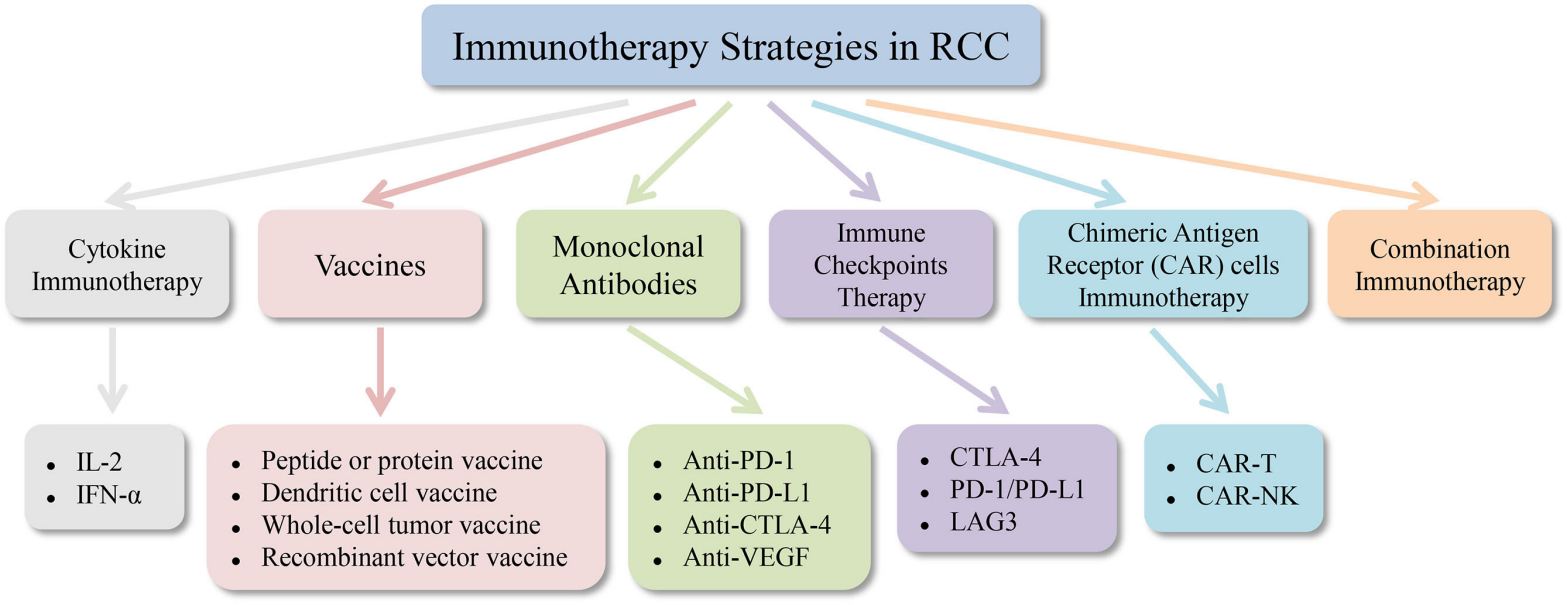
Immunotherapy strategies in renal cell carcinoma. RCC, renal cell carcinoma; IL-2, interleukin-2; IFN-α, interferon-α; PD-1, programmed cell death protein 1; PD-L1, programmed cell death protein ligand 1; CTLA-4, cytotoxic T lymphocyte antigen 4; VEGF, vascular endothelial growth factor; LAG3, gene 3 activation of lymphocyte; CAR, chimeric antigen receptor.

Interleukin-2 and interferon-α cytokine therapy have been used as a routine treatment for metastatic renal cell carcinoma (mRCC) (114). It has been stated in recent years that the use of anti-angiogenic drugs can improve the prognosis of RCC patients (115). However, targeted medications seldom achieve complete remission (CR), and most patients ultimately develop resistance, so discovering new methods for the treatment of renal cell carcinoma is a top priority. Like melanoma, renal cell carcinoma is also a minority of solid tumors with less susceptible to conventional cancer treatment approaches, such as chemotherapy and radiotherapy. Renal cell carcinoma tends to be a form of immune sensitive tumor, so many immunotherapy methods have been developed and various degrees of clinical outcomes have been achieved. Drug treatment for renal cell carcinoma is becoming more and more diversified from the era of cytokines to selective drug therapy and then to the era of immunotherapy. Combined immunotherapy has gained more and more attention and recognition in recent years. Figures 2, 3 are schematic diagrams of the history of development of RCC immunotherapy and treatment strategies.

### Vaccines

Cancer vaccines have the purpose of using unique and immunogenic tumor antigens to trigger, restore or reinforce the anti-tumor immune response of the body and to eradicate residual and metastatic tumor cells. Renal cell carcinoma is one of the most vulnerable solid tumors to immunotherapy and the vaccine technique is reasonably simple to adopt since it is an immunogenic tumor.

#### Peptide or Protein Vaccines

Polypeptide vaccines use tumor-associated peptides (TUMAPs) to trigger humoral and cellular immune responses, enhance the body's anti-cancer capacity, and prevent tumor growth, spread, and recurrence in order to suppress or regulate tumors (116). TUMAPs are peptides that occur mostly in tumor cells, but they do not occur in normal cells or tend to be limited. Most peptide or protein vaccines lack apparent immunogenicity and cannot cause a strong immune response when used alone. In conjunction with immune adjuvants (such as adjuvants targeting Toll-like receptors or cytokine adjuvants), peptide or protein vaccines are also typically used to improve the immune response (117).

IMA901 consists of nine HLA-I-binding TUMAPs that activate CD8^+^ CTL and one HLA-II-binding TUMAP that activates CD4^+^ helper T cells. Since IMA901 contains 10 different renal cell carcinoma-related peptides, it can induce a variety of T cell expansions against different epitopes, exert an immune response from T cells, and decrease the risk of immune escape in renal cell carcinoma. The OS rate of patients was correlated with T cell response to IMA901 in a phase II study of mRCC patients (118). A phase III multicenter, open-label, randomized study evaluated whether the addition of IMA901 to first-line sunitinib therapy can prolong OS in patients with metastatic or locally advanced RCC (119). In this study, 1,171 patients were screened, 339 of whom were randomized to receive sunitinib plus IMA901 (*n* = 204) or sunitinib monotherapy (*n* = 135). Vaccination with IMA901 plus granulocyte macrophage colony-stimulating factor in addition to first-line sunitinib did not prolong OS relative to sunitinib alone in patients with advanced, previously untreated metastatic renal cell carcinoma. Unlike the results of the Phase II study, the magnitude of the CD8^+^ T cell response is very low in the phase III study, which could be triggered by an adverse inhibition of the T cell activation induced by sunitinib or IMA901 or both. In summary, the IMA901 peptide vaccine administered with GM-CSF and single-dose cyclophosphamide demonstrated increased clinical benefit in patients with RCC. The rational use of adjuvants makes peptide vaccines more effective, and the combination of tumor vaccines and targeted therapies offers a promising approach to the treatment of renal cell carcinoma. Future research should concentrate on how to enhance the conditions for improving the OS.

Efficacy of autologous tumor-derived heat shock protein (glycoprotein 96)-peptide complex (HSPPC-96; vitespen) vaccine was assessed in a randomized phase III trial in patients at high risk of recurrence following resection of locally advanced renal cell carcinoma and there was no difference in recurrence-free survival (RFS) between patients treated with vitespen after nephrectomy and those not treated (120). The basic antigen G250 (carbonic anhydrase IX; CAIX) is expressed on the surface of 75% of RCC cells (90% of clear renal cell carcinoma) but has minimal expression in normal cells (121, 122), so that it can become one of the possible therapeutic targets. Tso et al. (123) identified a novel strategy for RCC vaccines that developed a fusion protein (FP) capable of delivering dual immune activators simultaneously: G250 and GM-CSF. The fusion protein GM-CSF-G250 obtained from the baculovirus expression vector system is a potent immunostimulant with the ability to activate immunomodulatory DCs and to induce T-helper cell-supported, G250-targeted and CD8^+^-mediated anti-tumor response. This completely suggests the efficacy of GM-CSF-G250 FP as an RCC cancer vaccine and can be used in clinical trials to treat advanced RCCs in the future.

#### Dendritic Cell Vaccines

**Figure 4:**
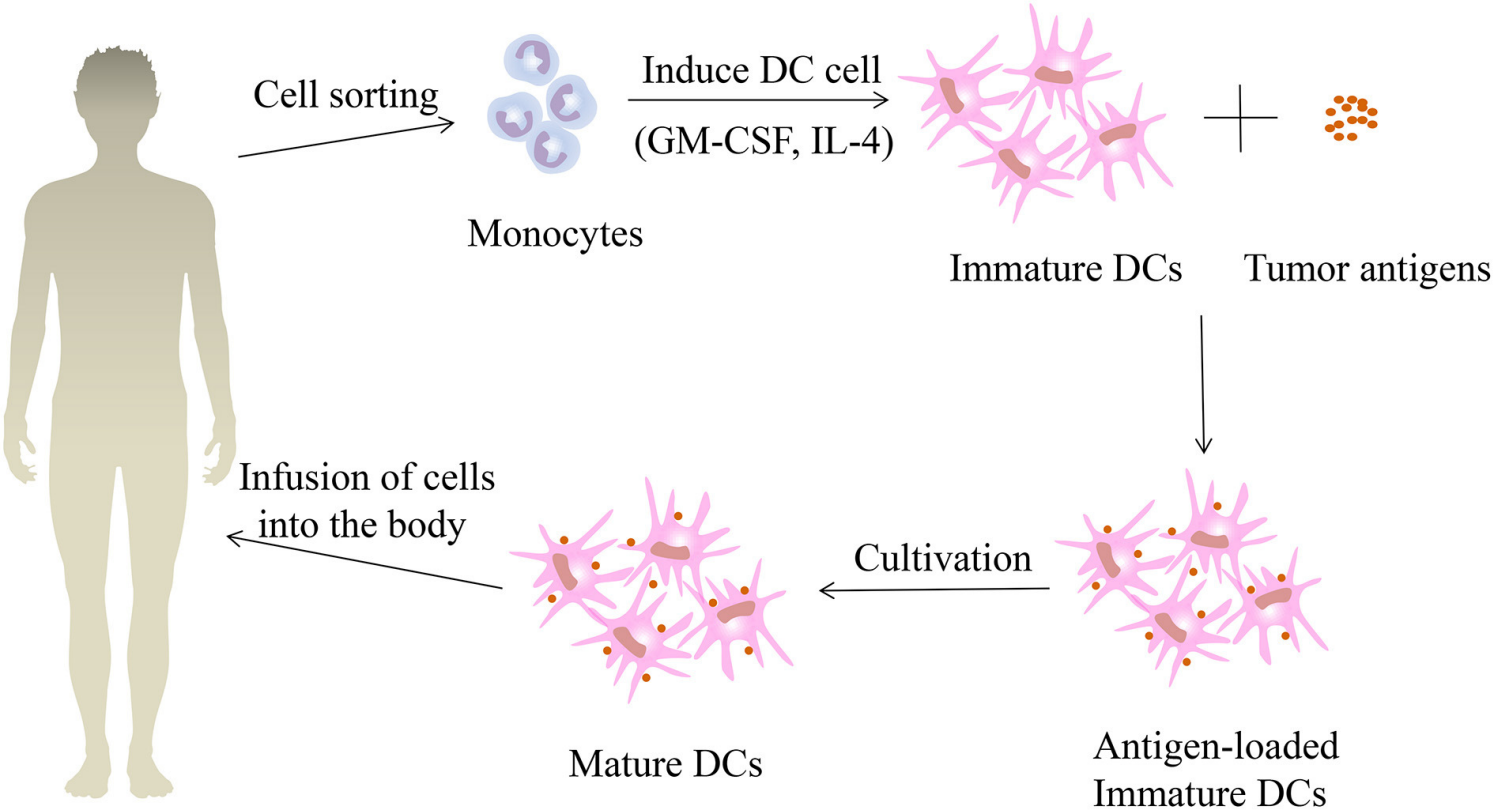
The treatment process of patients with renal cell carcinoma using dendritic vaccine. First, monocytes were collected and isolated from the peripheral blood of patients with renal cell carcinoma, and immature dendritic cells (DCs) were formed under the induction of granulocyte macrophage colony-stimulating factor (GM-CSF) and interleukin-4 (IL-4). Then the immature DCs are fused with the renal cell carcinoma tumor antigens to obtain immature DCs loaded with tumor antigens. After continuing the culture, the mature DC vaccines are obtained and then injected back into the renal cell carcinoma patients.

DCs are known to be a powerful antigen presenting cell in human body, and they are the initiator of anti-infection and anti-tumor immunity. Based on the strong immune properties of DCs, the DC vaccine has been established. The method of administering the DC vaccine to patients with renal cell carcinoma is shown in Figure 4. The DC vaccine offers a successful cure for renal cell carcinoma immunotherapy. AGS-003 is a new immunotherapy currently being developed for mRCC patients which introduces ribonucleic acid (RNA) into DCs derived from autologous mature monocytes from patient-specific tumors. It is an autologous DC vaccine that induces an immune response mediated by effector cells by presenting CD4^+^ and CD8^+^ T cells with unique epitopes (124). In an open-label phase II review, Amin et al. (125) assessed the efficacy of AGS-003 in combination with sunitinib in 21 mRCC patients at intermediate and poor risk. Results showed that 13 patients (62%) had clinical benefit and median progression free survival (PFS) was 11.2 months with median PFS 5.8 months for low risk patients and 19.4 months for intermediate risk patients. The median OS for all patients was 30.2 months, with an average median OS of 9.1 months for low risk and 61.9 months for high risk patients. This study showed that the combination of AGS-003 and sunitinib is well-tolerated, which can produce supportive immune responses and prolong survival in non-selected, intermediate and low risk mRCC patients. Based on the above clinical results, researchers have developed an international randomized phase III clinical trial (NCT01582672), namely autologous DC immunotherapy (AGS-003) plus standard therapy for advanced renal cell carcinoma (ADAPT) that still pending. In summary, AGS-003 combined with sunitinib substantially prolongs the survival of patients and has had no toxicity, with a better effect on the treatment of mRCC. If the phase III trial is successful, immunotherapy will dramatically boost the current status of renal cell carcinoma treatment.

DCs fuse with tumor cells to produce immunogenic hybrid cells, which can present a variety of tumor-related antigens based on the HLA molecule of DC. Avigan (126) injected autologous DC/tumor fusion vaccine into 23 patients (10 breast cancer patients and 13 renal cell carcinoma patients). The vaccine demonstrated high tolerance and no significant treatment-related toxicity. Immunological responses and disease regressions have been observed in some patients. Based on the above research, Avigan et al. performed a Phase I/II trial in 24 patients with stage IV renal cell carcinoma using allogeneic DC/tumor fusion vaccination (127). Vaccination resulted in antitumor immune responses in 10 patients as shown by an improvement in CD4^+^ and/or CD8^+^ T-cell expression of IFN-γ. Two patients had a partial therapeutic response and eight patients had a stabilization of their disease. The above findings indicate that autologous or allogeneic DC/tumor fusion vaccines have clinical importance in the treatment of patients with RCC. In addition, tumor vaccines can be developed by transfecting RNA-encoded DC antigens. Results of the phase I clinical trial showed that most patients with renal cell carcinoma immunized with autologous RNA transfected DC tumor had a vaccine-induced T cell response (128). This indicates that RNA-transfected DC immunization is a promising method to activate the body to develop successful anti-tumor immunity, which provides the basis for research into the treatment of renal cell carcinoma.

#### Whole-Cell Tumor Vaccines

Whole-cell tumor vaccines include autologous and allogeneic tumor cell vaccines. These tumor cells are inactivated by *in vitro* irradiation and maintain immunogenicity but without tumorigenicity. They are injected back into the host alone or combined with adjuvants to activate the body's anti-tumor response. There is no need for this approach to identify antigens. Autologous tumor cell vaccines offer the best antigen spectrum, but require a large number of tumors and a time-consuming and expensive preparation procedure. Allogeneic tumor cell vaccines can address the above problems well, but they do not contain antigens appropriate for specific patients, which are reasonably easy to prepare.

Schwaab et al. (129) performed a study in which a combination vaccine of autologous tumor cells and GM-CSF was administered to 22 patients with stage II to IV RCC pathology to determine the tumor-specific CD4^+^ and CD8^+^ T cell precursors associated with treatment and the results showed that tumor specific CD4^+^ (*P* = 0.028) and CD8^+^ (*P* = 0.018) T cell precursors increased significantly during treatment. In addition, it has been found that autologous tumor cell vaccine combined with IFN-α promotes immune activation and induces clinical response in metastatic renal cell carcinoma (130). Genetic engineering has been used on the basis of simple tumor cell vaccines to improve the immunogenicity of tumor cell vaccines to form genetically modified tumor cell vaccines. The method of action is to insert immunostimulatory cytokine or immune costimulatory molecule genes into tumor cells to enhance the induced immune response. Tani et al. (131) tested the clinical efficacy of GM-CSF-translated autologous renal tumor cell vaccine (GVAX) in four patients with stage IV renal cell carcinoma in Japan shown that GVAX greatly improved the immune antitumor and humoral responses of patients with renal cell carcinoma. Similarly, Antonia et al. (132) have transfected exogenous B7-1 gene to force the expression of RCC cells, which can increase the immunogenicity of tumor cells and then send costimulatory signals to T cells. The efficacy of B7-1 (CD-86) transduced autologous tumor cell vaccine combined with IL-2 was evaluated in a single center phase II trial for the treatment of stage IV renal cell carcinoma (133). In summary, methods involving cytokines and autologous tumor vaccines have demonstrated some clinical effectiveness and can be used as an adjuvant treatment following resection of renal cell carcinoma.

#### Recombinant Vector Vaccines

Recombinant vector vaccines may be divided into viral vector vaccines, bacterial vector vaccines, and plasmid DNA vaccines by different vectors. Tumor antigen 5T4 (trophoblast glycoprotein) is expressed in 95% of clear cell and papillary RCC specimens (134). Attenuated vaccine virus, adapted vaccine Ankara (MVA) has been developed to deliver 5T4 tumor antigen (TroVax). A phase II study examined the safety and clinical efficacy of MVA-5T4 alone or in combination with interferon-α (IFN-α) in patients with metastatic renal cell carcinoma, suggesting that MVA-5T4 was well-tolerated and immunogenic in mRCC patients when used alone or in combination with IFN-α (135). Another phase II trial tested the efficacy of TroVax in combination with IL-2 in 25 patients with mRCC (136). The results of this study showed that 21 patients had 5T4-specific antibody responses with two patients had a full response for more than 24 months and one patient had a partial response for more than 12 months. In addition, a phase III clinical trial tested the OS and protection of patients with sunitinib, IL-2, or IFN-α combined with MVA-5T4 (*n* = 365) or placebo (*n* = 368) in metastatic ccRCC. There was no substantial median OS difference between MVA-5T4 (20.1 months) and placebo (19.2 months; *P* = 0.55) (137). The above findings indicate that the magnitude of the 5T4-specific antibody response is positively associated with the survival time of patients, but the study failed to achieve the goal of improving the survival rate.

As the science and development of tumor vaccines continue to evolve and advance, more and more vaccines can be added to clinical care following experimental trials in the future. The goal of the vaccine is to increase the survival rate of patients with renal cell carcinoma and to reduce the risk of recurrence and metastasis. More and more focus has been given to TME and combination therapy in recent years, and RCC vaccines will have a wider prospect in the future.

### Cytokine Immunotherapies

#### Interleukin-2

The incidence of renal cell carcinoma is growing year by year, and for patients with advanced renal cell carcinoma, IL-2 is the first option. In 1992, the US Food and Drug Administration (FDA) approved high-dose interleukin-2 (HD IL-2) for the study of metastatic renal cell carcinoma based on a persistent response in a limited number of patients (138). In addition, studies have shown that in properly selected patients with metastatic renal cell carcinoma, HD IL-2 can produce a high response rate and lasting remission rate (139).

However, high doses of IL-2 can be associated with apparent toxicity. Capillary leakage syndrome, such as hypovolemic shock, renal ischemia, and pulmonary edema is primarily responsible for the toxicity of HD IL-2. Scientists have sought a low-dose IL-2 drug to reduce its toxicity. Vaglio et al. (140) found that long-term use of low-dose IL-2 and IFN-α is an effective and well-tolerated treatment choice that can prevent tumor metastasis and extend RFS in low-risk mRCC patients. In addition, the study showed that mRCC patients treated with low-dose IL-2 had a median OS of 19.8 months, while median PFS was 3.82 months, and that PFS was prolonged in patients treated with IL-2 as first-line or second-line treatment following IFN-α therapy (141). However, the Italian Oncology Community for Clinical Research (GOIRC) phase III randomized multicenter trial reported different results (142). No change in OS or RFS was observed in patients with renal cell carcinoma after surgery who obtained low-dose IL-2 and IFN-α adjuvants. The above results indicate that the order of administration of IL-2 and IFN-α may affect the survival of patients with renal cell carcinoma, and more rigorous and in-depth research is needed to explain the mechanisms of cytokine combination therapy for renal cell carcinoma.

As far as its toxicity is concerned, patients need to be hospitalized for monitoring, and the toxicity will generally disappear after drug withdrawal. Based on the above studies, it is urgent to determine the dose, route and combination therapy of IL-2 for the treatment of renal cell carcinoma. Future trials not only improve the remission rate, but also prolong the survival time.

#### Interferon-α

Interferon-α is now one of the most commonly used drugs in immunotherapy for patients with advanced renal cell carcinoma with biological effects, such as anti-virus, immune regulation and inhibition of tumor cell proliferation (143). Like IL-2, IFN-α therapy can also cause toxic reactions, such as fatigue, weakness, fever, chills and myalgia, depression, elevated transaminase, and autoimmunity (144). Flanigan et al. (145) found that patients with renal cell carcinoma treated with IFN-α-2b after nephrectomy had longer survival than interferon alone, indicating that nephrectomy before IFN-α treatment for metastatic renal cell carcinoma can bring significant survival benefits to patients. Several trials have evaluated the efficacy of IFN-α alone in the treatment of metastatic RCC (mRCC), with an average remission rate of about 15%. Because the remission rate of single drug IFN-α is not high, the researchers have chosen to combine it with IL-2 and findings showed that the remission rate of IL-2 combined with IFN-α-2a was 18.6%, and the 1-year disease-free survival rate was 20%. The remission rate of IFN-α-2a alone was only 7.5%, and the 1-year disease-free survival rate was only 12% (146). This indicates that IL-2 and IFN-α have anti-tumor activity in renal cell carcinoma, and the triple immunotherapy that adds 5-fluorouracil to enhance the clinical therapeutic effect (147). The above phenomena suggest that the anti-tumor effect of IFN-α is very limited, and IFN-α does not seem to be suitable as a single drug for the treatment of renal cell carcinoma.

The effect of cytokine therapy on renal cell carcinoma has not yet been positive, the survival time has not been long, and the occurrence of toxic reactions is inevitable, however, patients have improved their remission rate. The era of cytokine therapy has passed and the use of selective drugs has shown a positive response in the clinical treatment of renal cell carcinoma.

### Monoclonal Antibodies

#### Anti-PD-1 Antibodies

##### Nivolumab

Nivolumab is a PD-1 monoclonal antibody that can block the interaction of PD-1 with its PD-L1/PD-L2 ligand, which enhances T cell function and *in vitro* exerts anti-tumor activity. Some studies have shown that there are differential expressions between primary and metastatic renal cell carcinoma of PD-1, PD-L1 and PD-L2 with shorter OS for primary or metastatic patients with overexpression of PD-1 (148). This means that PD-1 expression is linked to poor prognosis, and the antibody's therapeutic activity against PD-1 can be linked to whether or not the tumor is metastasized. The choice of personalized immunotherapy for renal cell carcinoma is offered if the relationship between them can be clarified.

A phase II clinical trial of nivolumab in metastatic renal cell carcinoma has been conducted (149). The results suggested that three doses of nivolumab (0.3, 2, 10 mg/kg) demonstrated controllable and healthy anti-tumor activity in patients who had been pre-treated with VEGF-targeted drugs. The most common treatment-related adverse effect (AE) was fatigue and the above results provided evidence for the phase III trial. The phase III clinical trial for nivolumab and everolimus is CHECKMATE 025 (150). In this study, 821 patients with mRCC who received antiangiogenic therapy were divided into two groups: 3 mg/kg of nivolumab every 2 weeks or 10 mg of everolimus once daily. The outcomes showed that the median OS for the nivolumab and everolimus groups was 25.0 and 19.6 months, respectively. Nineteen percent of patients treated with nivolumab and 37% of patients treated with everolimus had treatment-related grade 3 or 4 AEs, and nivolumab AE was the same in phase II and phase III trials. The above results indicate that in clinical treatment of renal cell carcinoma, nivolumab is more effective than everolimus.

##### Pembrolizumab

Another humanized monoclonal anti-PD-1 antibody is pembrolizumab, which has been widely studied in multiple malignant tumors (151–153). The efficacy of axitinib in combination with pembrolizumab in patients with advanced renal cell carcinoma was evaluated in a non-randomized, open-label phase Ib trial (154). The study showed that, in untreated patients with advanced renal cell carcinoma, the combination of axitinib and pembrolizumab was safe and tolerable and had anti-tumor efficacy, but the molecular mechanism of the possible synergistic effect is still unclear. In a recent open-label phase III clinical trial (KEYNOTE-426), Rini et al. (155) investigated whether pembrolizumab in combination with axitinib achieved better outcomes in patients with advanced renal cell carcinoma previously untreated than sunitinib. The findings showed that the number of patients in the pembrolizumab-axitinib group who lived for 12 months was 89.9 and 78.3% in the sunitinib group (*P* < 0.0001). In the pembrolizumab-axitinib group, median progression-free survival was 15.1 months, and in the sunitinib group, 11.1 months (*P* < 0.001). In the pembrolizumab-axitinib group, the objective remission rate (ORR) was 59.3% and the ORR was 35.7% in the sunitinib group (*P* < 0.001). In summary, pembrolizumab-axitinib treatment significantly extended OS and PFS compared with sunitinib alone, and significantly improved ORR in patients with advanced renal cell carcinoma who had not undergone prior therapy. The results of this study indicate that the use of anti-VEGF immunotherapy alone does not appear to be as successful as combination therapy alone, and the prospects for the potential use of the combination of immunotherapy would be quite broad.

#### Anti-PD-L1 Antibodies

##### Atezolizumab

Atezolizumab is a human IgG1 monoclonal antibody targeting PD-L1 that aims to interfere with the binding of PD-L1 and its two receptors, PD-1 and B7-1 (156). By blocking the immune checkpoint PD-1/PD-L1, atezolizumab decreases the immunosuppressive signal in TME, thereby improving T-cell-mediated anti-tumor immunity. In the phase I trial, 70 patients with mRCC (clear renal cell carcinoma, *n* = 63 and non-clear renal cell carcinoma, *n* = 7) received intravenous atezolizumab injection every 3 weeks (157), showing that the median OS of patients with clear renal cell carcinoma was 28.9 months, the median progression-free survival period was 5.6 months, and the ORR was 15%. These findings indicate that atezolizumab has demonstrated good protection and promising anti-tumor activity in patients with mRCC.

##### Avelumab

Human monoclonal antibody avelumab directly affects the relationship between PD-1 and PD-L1 against PD-L1 and spreads of tumor cells via antibody-dependent cell-mediated cytotoxicity (158). The advantage of antibody-dependent cytotoxic-mediated cells in immunotherapy for immune checkpoints can improve the therapeutic impact. JAVELIN Renal 100 is an ongoing open-label, multicenter phase Ib trial in 14 centers in the United States, the United Kingdom and Japan (159). The study demonstrated the safety and anti-tumor activity of avelumab combined with axitinib in untreated patients with advanced renal cell carcinoma and was consistent with the characteristics of a single drug alone. A subsequent phase III trial measured the difference in efficacy between avelumab combined with axitinib and sunitinib alone (160). In 560 patients with PD-L1 positive renal cell carcinoma (63.2%), the median progression-free survival of avelumab plus axitinib was 13.8 months, while the median progression-free survival of sunitinib was 7.2 months. The ORR of avelumab together with axitinib was 55.2% and the ORR of sunitinib was 25.5%. The rate of adverse reactions in both groups was more than 99%, most likely due to two VEGF inhibitors, axitinib and sunitinib. If the frequency of adverse effects can be minimized by increasing the dosage of drugs or using other approaches, combination immunotherapy can significantly improve the benefits for patients.

#### Anti-CTLA-4 Antibodies

##### Ipilimumab

CTLA-4, an inhibitory receptor expressed on the surface of T cells that can bind to CD80 and CD86, blocks normal T cell proliferation and plays a biological role (161). It plays a key role in controlling immune responses to tumors and is considered a possible cancer immunotherapy target. Ipilimumab is an anti-CTLA-4 (IgG1) antibody that can effectively prevent CTLA-4 from binding to the ligand, thus activating T cells. A phase II single-drug clinical trial of ipilimumab in patients with metastatic renal cell carcinoma found that 5 out of 40 partial response patients and 33% of patients had immune-mediated grade 3 or 4 toxicity, most frequently seen in enteritis and pituitary gland disease. The link between AE and tumor regression was highly significant (162). This also indicates that T cell control will influence the progression of the tumor and then treat patients with carcinoma of the renal cells. Ipilimumab may be used alone or in conjunction with vaccines or other antibodies in immunotherapy for renal cell carcinoma. The CHECKMATE 016 research assessed the efficacy and protection in patients with metastatic renal cell carcinoma of nivolumab combined with ipilimumab (163). The study found that 42.1 and 36.8%, respectively, were the ongoing responses of patients in the N3I1 and N1I3 arms, and the 2-year OS was 67.3 and 69.6% in the N3I1 and N1I3 arms, respectively. In 38.3 and 61.7% of patients in the N3I1 and N1I3 arms, respectively, grade 3–4 treatment-related AEs were registered. While the two combination therapy groups referred to above are successful, N3I1 appears to be more appropriate for clinical use because the AE of the N3I1 group is lower. The efficacy of nivolumab plus ipilimumab vs. sunitinib was evaluated in a recent randomized phase III trial based on N3I1 (CHECKMATE 214) (164). It was found that nivolumab plus ipilimumab improved OS compared to sunitinib in patients at moderate or low risk, who had previously untreated advanced renal cell carcinoma, who had previously induced less AEs, and who had a higher health-related quality of life (HRQoL). This clearly suggests that the effectiveness of combination therapy with nivolumab-ipilimumab is greater than that of sunitinib, which is correlated with HRQoL improvement. The combination of monoclonal antibodies is a novel immunotherapy that affects the proliferation and function of T cells and other immune cells at different immune control points, but there is a need for more studies to validate their role in the treatment of carcinoma of renal cells.

#### Anti-VEGF Antibodies

##### Bevacizumab

As mentioned above, VEGF derived from tumors can affect immune cell activity and cause tumors to escape immune surveillance, so anti-VEGF antibodies may boost T cell infiltration in TME and destroy tumors through vascular normalization and activation of endothelial cells. Bevacizumab, an anti-VEGF antibody, tested the combination of interferon-α and bevacizumab in metastatic renal cell carcinoma, resulting in substantially longer progression-free survival relative to interferon-α alone makes it approved for a first-line treatment of metastatic renal cell carcinoma (165). Studies have shown that CD8^+^ T cells, MHC-1, Th1, T-effector markers and chemokines, most notably C-X3-C motif chemokine ligand 1 (CX3CL1), increase in metastatic renal cell carcinoma in patients treated with atezolizumab and bevacizumab (166). The above data indicate that the combination of anti-VEGF antibody and anti-PD-L1 antibody can boost the migration of antigen-specific T cells, but the underlying specific mechanism needs to be studied in order to elucidate. A multicenter randomized phase III study (IMmotion151) assessed the efficacy of atezolizumab plus bevacizumab vs. sunitinib in patients with previously untreated metastatic renal cell carcinoma (167). The median progression-free survival of the PD-L1 positive population was 11.2 months in the atezolizumab plus bevacizumab group and 7.7 months in the sunitinib group (*P* = 0.027). Forty and 54% of patients developed grade 3–4 AE-related therapies, respectively. Although there was no statistical difference in progression-free survival between the two groups compared to sunitinib, atezolizumab plus bevacizumab could extend the progression-free survival of patients with metastatic renal cell carcinoma and demonstrate good safety. The above findings endorse atezolizumab plus bevacizumab as a first-line treatment option for some patients with advanced renal cell carcinoma.

### Immune Checkpoint Therapies

The application of an immune checkpoint inhibitor has opened up a broad prospect for renal cell carcinoma immunotherapy in recent years. Under normal conditions, the body's immune checkpoints will preserve the normal immune microenvironment by controlling the autoimmune response, but when the tumor arises, the tumor cells trigger the immune checkpoint abnormally, so body's immune system is unable to remove tumor cells in time, leading to an immune escape from the tumor. Inhibitors of the immune checkpoint can reactivate immune cell signal transduction, control immune response, and then destroy tumor cells. The immune checkpoint inhibitor currently includes drugs targeting CTLA-4, PD-1, and gene 3 activation of lymphocyte (LAG3).

One of the checkpoints in the immune response process is CTLA-4, which is an inhibitory receptor on the surface of T cells. On the surface of tumor cells, CTLA-4 and CD28 compete with B7-1/2 to inhibit and activate T cells, respectively. *CTLA-4* knockout mice have been reported to play an important role in down-regulating T cell activation and maintaining homeostasis of lymphocytes. These mice develop multi-organ lymphocyte infiltration-associated lymphoproliferative diseases, culminating in death at 3–4 weeks of age (168). The monoclonal antibody ipilimumab has shown good efficacy in the treatment of carcinoma of renal cells, but active intervention is still needed for the incidence of severe immune-related AEs.

Furthermore, PD-1 is an immune checkpoint receptor and has become an anti-tumor immunotherapy target. On activated effector T cells, NK cells, and B cells, PD-1 expressed. Nivolumab and pembrolizumab monoclonal antibodies are immune blockers targeting PD-1 and providing renal cell carcinoma patients with long-lasting reactivity.

LAG3 is an immune checkpoint for T cell activity and its main ligand is the major complex of histocompatibility II (169). Recent studies have shown that *LAG3* DNA methylation is significantly associated with LAG3 expression of tumor cells and immune cell infiltration in clear renal cell carcinoma (170). This shows that DNA methylation can achieve epigenetic control of LAG3 in tumors and immune cells, which also provides support for LAG3 as an immune checkpoint.

Although immune checkpoint inhibitor therapies have shown better results in the improvement of OS in ccRCC, the efficacy of anti-PD-1/PD-L1 agents in patients with non-clear RCC (nccRCC) and other unusual tissue subtypes of RCC remains controversial. Due to the limited number of these ICI-treated patients, it is not possible to reliably determine the role of anti-PD-1/PD-L1 agents in this population. A multicenter pooled review offers insights into the clinical treatment of nccRCC and sarcomatoid/rhabdoid RCC patients, demonstrating the differential activity of PD-1/PD-L1 blockers in patients with different RCC histologies (171). It is hoped that more relevant clinical trials will be performed in the future to improve results for nccRCC patients.

### Cellular Immunotherapy Modified by Chimeric Antigen Receptor

Chimeric antigen receptor modified immune effector cells (CAR-T and CAR-NK) therapies are newly developed cancer adoptive therapies. CAR-T cell therapy has displayed remarkable clinical responses in multiple malignant hematological tumors in recent years (172). Lamers et al. (173) tested the protection of first-generation CAIX-CAR-T cells in patients with metastatic renal cell carcinoma and found that circulating CAR-T cells could be identified in all patients and retained antigen-specific immune function after treatment, but patients developed anti-CAR-T cells and cellular immune responses and liver enzyme disorders triggered by CAR-T. Recently, cytokine release syndrome and immune cell-associated neurological syndrome have been reported to be toxic to CAR-T, although the incidence of acute kidney injury is relatively low and most patients will recover within 30 days suggesting that the therapeutic impact of CAIX-CAR-T cell therapy on renal cell carcinoma is limited (174).

In order to find a stronger therapeutic effect, the researchers used CAR-T cells in conjunction with chemotherapy agents and findings showed that the second-generation CAIX-CAR-T cells plus sunitinib had synergistic efficacy against the model of human renal cell carcinoma in the mouse lung (175). This suggests that the combination of CAIX-CAR-T and sunitinib may induce an effective anti-tumor response in experimental models of metastatic renal cell carcinoma. In addition, Zhang et al. (176) used a lentiviral vector to transduce CAIX-specific third-generation CAR into NK92 cells and evaluated the immune effect of CAR-NK92 cells against CAIX-positive RCC cells *in vitro*, and found that the cytotoxicity of CAR-NK92 cells was enhanced after RCC cells were treated with bortezomib. The results indicate that the combined strategy may be a possible therapy to improve adoptive CAR-T and CAR-NK92 cell immunotherapy, which may be applied in the future to autologous or allogeneic T cells or NK cells and may provide more care for RCC.

### Combination Immunotherapies

The combination immunotherapy of renal cell carcinoma has had important clinical benefits for RCC patients. In a phase I/II clinical trial, the combination of monoclonal antibody WX-G250 (RENCAREX®) and low-dose IFN-α-2a demonstrated good protection and tolerance with lower toxicity in patients with metastatic renal cell carcinoma (177). Subsequent trials have also shown that WX-G250 is an efficient combination partner and could be used in combination with other drugs to test for efficacy in the future. More and more studies have shown that combination clinical studies using immune checkpoint antibodies and inhibitors that block the VEGF pathway have become a new standard for the care of advanced RCC patients. The immunomodulatory properties of anti-angiogenesis therapies combined with immune checkpoint inhibitors can enhance clinical therapeutic effects through a variety of mechanisms of action. As indicated in the previous section, the combination of monoclonal antibody pembrolizumab and axitinib significantly extended OS and progression-free survival and significantly improved ORR and similar findings were observed in combination with avelumab and axitinib. In addition, the combination therapy of nivolumab plus ipilimumab and atezolizumab plus bevacizumab also demonstrated substantial clinical efficacy. The combination of immunotherapy and chemotherapy is also an integral part of the treatment of renal cell carcinoma. Due to the variety of immunotherapy for renal cell carcinoma, the combination of various therapies will enhance the body's anti-tumor immunity and reduce the occurrence of AEs. Combination therapy is intended to improve effectiveness but not increase toxicity in a broader variety of patients with renal cell carcinoma. In addition, the drug resistance phenomenon in the treatment of renal cell carcinoma can be minimized or overcome by combination therapy, so there is an urgent need to find the best and easiest combination approach, and related clinical studies are ongoing.

## Conclusions and Perspectives

In recent years, as the molecular biology of renal cell carcinoma continues to advance, immune escape mechanisms for renal cell carcinoma, including MHC, immunosuppressive cells and their secreted immunosuppressive cytokines, signal transduction of apoptotic molecules have been explored. It is especially concerned that the immune microenvironment of the tumor has a major impact on immune escape, but the immune microenvironment is very complex and evolving, and targeting one of these compounds can trigger chain changes. This involves the collective intervention of multiple aspects to alter the microenvironment of the tumor immunosuppressive to achieve the goal of killing or even removing the tumor. Advances in glycobiology and immunology have now shown that improvements in the sugar chain and glycosylation provide a selective advantage for tumor cells to avoid anti-tumor immunity and even to engage in the modulation of immune cell function to encourage tumor growth (178). Throughout literature, the correlation between changes in the sugar chain and immune escape from renal cell carcinoma has not been documented, which would be a new field to be explored, and understanding the impact of tumor-derived glycans on the molecular immune system would improve the strategy of immunotherapy for renal cell carcinoma.

Identifying the immune escape mechanisms of renal cell carcinoma has laid a solid foundation for identifying suitable molecular targets, thus dramatically enhancing the clinical effectiveness of RCC treatment from the period of systemic cytokine administration to the era of targeted drugs and then to the era of immunotherapy. Table 2 summarizes the phase III immunotherapy-based clinical trials of renal cell carcinoma and the outcome of ongoing trials will assess the trend of care for RCC. It is hoped that immunotherapy will dramatically increase patients' survival rates in the future and reduce the occurrence of AEs by using less doses of drugs. New immunotherapy is an essential complement to RCC therapy, but several problems remain, such as the safety of drugs, including the mixture of drugs, the sequence of drugs, the dosage of drugs, the stage of treatment, and so on. The choice of immunotherapy targets for patients is also very important, and the effectiveness of drugs at the same target can differ from person to person. The opening of the precision medical model and the creation of individual treatment plans would offer different treatment experiences to patients with renal cell carcinoma, thereby enhancing clinical efficacy. Preclinical trials and animal models therefore play a very important role in the optimization of RCC immunotherapy. Meanwhile, future studies could explore molecular markers that can control immune efficacy to direct the treatment of renal cell carcinoma.

**Table 2:** Ongoing phase III clinical trials based on immunotherapy in renal cell carcinoma.

**Patient population**	**Intervention/Treatment**	**Targets**	**Enrollment**	**Recruitment status**	**NCT number**
Renal cell carcinoma	Avelumab (MSB0010718C) Axitinib (AG-013736) Sunitinib	PD-L1	886	Active, not recruiting	NCT02684006
Renal cell carcinoma	Lenvatinib Everolimus Pembrolizumab Sunitinib	PD-1	1,069	Active, not recruiting	NCT02811861
Renal cell carcinoma	Nivolumab Cabozantinib Sunitinib Ipilimumab	PD-1/CTLA-4	638	Active, not recruiting	NCT03141177
Renal cell carcinoma	Pembrolizumab Placebo (saline solution)	PD-1	950	Active, not recruiting	NCT03142334
Renal cell carcinoma	Atezolizumab Placebo	PD-L1	778	Active, not recruiting	NCT03024996
Renal cell carcinoma	Atezolizumab (MPDL3280A) Bevacizumab Sunitinib	PD-L1	915	Active, not recruiting	NCT02420821
Renal cell carcinoma	Pembrolizumab Axitinib Sunitinib	PD-1	861	Active, not recruiting	NCT02853331
Renal cell carcinoma	Pembrolizumab Epacadostat Sunitinib Pazopanib	PD-1	129	Active, not recruiting	NCT03260894
Renal cell carcinoma	Nivolumab Ipilimumab Nivolumab placebo	PD-1/CTLA-4	1,600	Recruiting	NCT03138512
Renal cell carcinoma	Nivolumab Ipilimumab Ipilimumab placebo	PD-1/CTLA-4	418	Recruiting	NCT03873402
Renal cell carcinoma	Cabozantinib Nivolumab Ipilimumab Cabozantinib-matched Placebo	PD-1/CTLA-4	676	Recruiting	NCT03937219
Renal cell carcinoma	Durvalumab Tremelimumab	PD-L1/CTLA-4	1,750	Recruiting	NCT03288532
Renal cell carcinoma	Atezolizumab Cabozantinib	PD-L1	500	Recruiting	NCT04338269
Renal cell carcinoma	Avelumab	PD-L1	137	Recruiting	NCT03815643
Metastatic renal cell carcinoma	Bempegaldesleukin Sunitinib Nivolumab Cabozantinib	PD-1	600	Recruiting	NCT03729245
Metastatic renal cell carcinoma	Nivolumab Ipilimumab	PD-1/CTLA-4	400	Recruiting	NCT03977571
Metastatic renal cell carcinoma	Nivolumab Patient Observation	PD-1	805	Recruiting	NCT03055013
Advanced or metastatic renal cell carcinoma	Nivolumab Ipilimumab Sunitinib	PD-1/CTLA-4	1,390	Active, not recruiting	NCT02231749
Advanced or metastatic clear-cell renal cell carcinoma	Nivolumab Everolimus	PD-1	1,068	Active, not recruiting	NCT01668784
Clear cell renal cell carcinoma	Cabozantinib Ipilimumab Nivolumab	PD-1/CTLA-4	1,046	Recruiting	NCT03793166
Renal cancer	Continue PD-1/PD-L1 Inhibitors Discontinue PD-1/PD-L1-1 inhibitor	PD-1/PD-L1	578	Recruiting	NCT04157985
Renal cancer	Checkpoint inhibitor (CPI)		200	Recruiting	NCT03755739

*Date extracted from https://www.clinicaltrials.gov. NCT, nationall clinical trial; PD-1, programmed cell death protein 1; PD-L1, programmed cell death protein ligand 1; CTLA-4, cytotoxic T lymphocyte antigen 4; CPI, checkpoint inhibitor*.

## Author Contributions

YJ, KY, and XS participated in the writing and revision of the manuscript. SW and DY provided the original concepts and were in charge of the final version of the manuscript. YJ and KY did the literature search and drew figures. XS and AA polished the manuscript and improved the English quality of the manuscript. JZ, KH, CX, ZX, HW, QX, and LZ revised the writing of the manuscript. All authors were involved in the conception, preparation of the manuscript, and the final version of the manuscript has been read and approved by all the authors before its submission.

## Conflict of Interest

The authors declare that the research was conducted in the absence of any commercial or financial relationships that could be construed as a potential conflict of interest.

## Abbreviations

RCC: renal cell carcinoma
CAR: chimeric antigen receptor
MHC: major histocompatibility complex
ccRCC: clear cell RCC
pRCC: papillary RCC
chRCC: chromophobe RCC
uRCC: unclassified RCC
ICI: immunocheckpoint inhibitor
HLA: human leukocyte antigen
TAP: transport proteins associated with antigen processing
LMP: low molecular weight protein
NK: natural killer cell
IFN-γ: interferon-γ
IL-10: interleukin-10
PGE2: prostaglandin E2
TGF-β: transforming growth factor-β
VEGF: vascular endothelial growth factor
DC: dendritic cell
COX: cyclooxygenase
Tregs: regulatory T cells
Bregs: regulatory B cells
MDSCs: myeloid-derived suppressor cells
TAMs: tumor-associated macrophages
IDO: indoleamine 2,3-dioxygenase
TME: tumor microenvironment
PMN-MDSC: polymorphonuclear MDSC
CTLA-4: cytotoxic T lymphocyte antigen 4
mRCC: metastatic RCC
TUMAPs: tumor-associated peptides
OS: overall survival
RFS: recurrence-free survival
CAIX: carbonic anhydrase IX
FP: fusion protein
RNA: ribonucleic acid
PFS: progression free survival
MVA: modified vaccinia Ankara
CR: complete remission
AE: adverse event
ORR: objective remission rate
LAG3: gene 3 activation of lymphocyte
nccRCC: non-clear cell RCC.
